# The Tele.TAnDem intervention: study protocol for a psychotherapeutic intervention for family caregivers of people with dementia

**DOI:** 10.1186/s12912-015-0059-9

**Published:** 2015-03-08

**Authors:** Renate Soellner, Maren Reder, Anna Machmer, Rolf Holle, Gabriele Wilz

**Affiliations:** 1 University of Hildesheim, Universitätsplatz 1, Hildesheim, 31141 Germany; 2 Helmholtz Zentrum München, German Research Center for Environmental Health, Ingolstädter Landstraße 1, Neuherberg, 85764 Germany; 3 Friedrich-Schiller-University Jena, Fürstengraben 1, Jena, 07743 Germany

**Keywords:** Dementia, Telephone-based psychotherapy, Family caregivers

## Abstract

**Background:**

Family caregivers are confronted with high demands creating a need for professional support and at the same time hindering its utilization. Telephone support allows easier access than face-to-face support because there is no need to leave the person with dementia alone or find an alternative carer. It is also independent of transport possibilities or mobility. The objectives are to evaluate whether telephone-based cognitive-behavioral therapy, which is implemented in established care provision structures, improves outcomes compared to usual care and whether it is as effective as face-to-face cognitive-behavioral therapy.

**Methods/Design:**

If participants live in the area of one of the study centers (Jena, Berlin, Munich) and indicate that attendance of a face-to-face therapy is possible, they will be assigned to the face-to-face group. The other participants will be randomized to receive either telephone-based cognitive-behavioral therapy or usual care. Data will be collected at baseline, post-intervention, and at a 6-month follow-up. The primary outcomes will be depressiveness, burden of care, health complaints, and problem-solving ability. The secondary outcomes will be anxiety, quality of life, violence in caregiving, utilization of professional assistance, and cost effectiveness.

**Discussion:**

This paper describes the evaluation design of our telephone-based cognitive-behavioral therapy in a randomized controlled trial. If this intervention proves to be an effective tool to improve outcomes, it will be made accessible to the public and the use of this support service will be recommended.

**Trial registration:**

German Clinical Trials Register DRKS00006355.

## Background

Numerous studies have shown that family caregivers, particularly those who care for a relative with dementia, face extremely burdensome demands and life changes, which impacts their health and quality of life. Increased morbidity [1] and mortality rates [2] and higher prevalence rates of anxiety and depressive disorders [3] are reported. These consequences are well documented and indicate a strong need for adequate professional support.

For family caregivers, accessing professional support is often difficult and existing support services are used insufficiently. Caregivers often need to monitor the person with dementia for most of the day. Especially elderly caregivers may themselves suffer from decreased mobility. Additionally, a large proportion of this group lives in rural areas where personal support services can be hard to reach. Therefore, the challenges family caregivers face create a special need for professional support and, at the same time, can hinder its utilization.

A possible solution is telephone-based support. It allows easier access than face-to-face support because there is no need to leave the person with dementia alone or find an alternative carer. It is also independent of transport possibilities or mobility. A telephone-based short-term intervention for family caregivers of people with dementia (Tele.TAnDem: a cognitive-behavioral telephone-based intervention for family caregivers of people with dementia that can also be delivered face-to-face) was already tested for feasibility, efficacy, and acceptability in a previous study [4].

In the present study, Tele.TAnDem will be implemented in established care provision structures (Alzheimer Society) for the first time. The aim of our study is to assess the effectiveness of this integrated intervention in a randomized controlled trial. The primary objective is to evaluate whether telephone-based cognitive-behavioral therapy (TEL) improves depressiveness, burden of care, health complaints, and problem-solving ability compared to usual care. The secondary objectives are to evaluate whether (1) TEL is as effective as face-to-face cognitive-behavioral therapy (F2F), (2) a two-day training program (8h) in Tele.TAnDem for behavioral therapists is sufficient to achieve an adequate and effective implementation of the intervention, (3) the TEL intervention is a cost-effective or possibly even cost-saving alternative compared to usual care, and (4) TEL increases the use of professional support by family caregivers of people with dementia compared to the control group.

## Methods/Design

### Design

**Figure 1:**
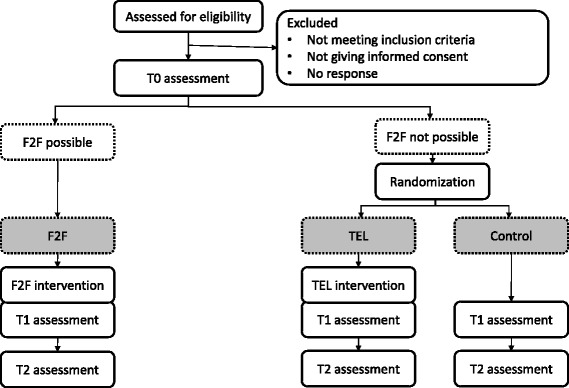
**Trial flow diagram.**

This study is a non-blinded two-armed parallel randomized controlled trial with a third non-randomized group. Participants will be asked whether they reside in the area of a study center and can participate in the F2F. If this is the case, they will be assigned to the F2F group. If this is not the case, they will be randomly assigned to the TEL group or usual care. Random allocation will be on a 1:1 ratio basis but separated for gender and will take place after the baseline assessment. All participants will receive information materials on care and dementia. Assessments will be conducted at baseline (T0), post intervention (T1) and a 6-month follow-up (T2) (Figure 1). The TEL group will receive 12 psychotherapy sessions on the phone, whereas the F2F group will receive 12 psychotherapy sessions in direct contact in one of the participating treatment centers.

### Participants’ eligibility

For the current study, we will recruit caregiving partners, children, and children-in-law. Inclusion criteria are that the caregiving relative has key responsibility for the care of the person with dementia and that the person with dementia has at least low grade dementia according to medical diagnosis. Exclusion criteria are that the caregiving relative receives ongoing psychotherapeutic treatment, has a severe physical illness or medically diagnosed psychiatric disorder, and that the person with dementia is institutionalized or institutionalization is planned for the next 6 months.

### Recruitment

The recruitment of the study participants will be nationwide with a special focus on Berlin, Jena and Munich, as the F2F intervention will only be performed in these centers. For recruitment, different public relations methods will be used: printed information materials available at cooperation partners (clinics, practices, home support services), editorial articles in regional and national newspapers, television and radio interviews, a project homepage (www.teletandem.uni-jena.de), and presentations of the study in groups for family caregivers.

### Ethical approval

Ethical approval was obtained from the Ethics Commission of the Friedrich-Schiller-University Jena. Participants will be informed about the content, purpose, and procedure of the study, and written informed consent will be obtained. The person with dementia will also receive information on the study. An indirect positive effect is expected on the well-being of the persons with dementia. In the previous study, the intervention did not pose any risks. No negative impact on the study participants is expected, since in case of severe depression or a suspected suicide intention, the caregiving relatives will be transferred to the institutions of usual care. Personal data will be stored separately from research data. The first, second, and third assessment (T0, T1, and T2) will be linked to each other by means of a researcher generated identification code. All personal data will be deleted after completion of the study. Participation in the study will be voluntary and the participants may withdraw consent at any time.

### Procedure

Once potential participants contact the study center, they will be screened for meeting the eligibility criteria in a manual-based telephone interview. Additionally, information on the participants’ sex as well as the possibility of taking part in F2F will be assessed in this screening.

After a positive screening, all potential study participants will receive an information package including information about the study, informed consent forms for study participation and data processing as well as for telephone contact, information for the person with dementia, and several leaflets on dementia and caregiving.

For the first assessment, participants will be contacted by phone, an appointment for the telephone interview will be made for about two weeks later, and the questionnaires will be sent out. This survey technique is used because a mostly elderly study population is expected. In addition, the acceptance of the survey is to be improved and the risk of missing data or dropout to be reduced. In the telephone interview, comprehension difficulties and unanswered questions will be discussed and the questionnaires will be reviewed for completeness. After the telephone interview, the questionnaires will be returned in a return envelope.

Participants will be allocated to the three study groups after finishing the baseline assessment (T0). To ensure a sufficient number of caregivers taking part in F2F, all participants living in Berlin, Jena or Munich for whom attendance is possible will be allocated to the F2F intervention, as long as their mobility is given. A bias is not expected because the participants will not be aware of being exclusively allocated to F2F, as they will be informed about being randomized to one of the three groups. The random number generator Random.org will be used for randomization. After being informed about their group allocation, participants in the intervention groups will receive the therapy sessions. After 6 months, when the therapy is finished, the T1 assessment will be conducted. After another 6 months, the T2 assessment will be conducted. The control group will receive an allowance for participation of 40 EUR, which will be transferred after completion of the study.

### Tele.TAnDem intervention

Members of both TEL and F2F will receive twelve 50-minute sessions of an individual cognitive-behavioral therapy (CBT) provided by trained psychotherapists within 6 months. The first four sessions will take place at weekly intervals and six further sessions will follow at fortnightly intervals. The two last sessions will be conducted at monthly intervals. Both TEL and F2F sessions will be offered by the same therapists to minimize person effects.

The theoretical concept of the intervention is based on the principles and methods used in CBT. The therapy strategies have been especially adapted for caregivers of people with dementia. The standardized and manual-based intervention [5] consists of 10 different therapy modules which can be used and combined by the therapist according to the individual needs of each participant:

1. *Basic elements.* Basic elements cover creating therapeutic alliance, structuring of each session and handling of crises.
2. *Problem analysis.* An individual problem analysis to identify the participants’ main problem areas in the caregiving situation and individual therapy goals is conducted.
3. *Psychoeducation.* The therapist provides information on dementia and its course to help participants handle the caregiving situation as well as supporting them in accepting the consequences of dementia.
4. *Strengthening problem solving abilities.* Through problem-solving training, participants are instructed and supported in their individual problem-solving process.
5. *Changing dysfunctional cognitions.* Through Socratic dialog and guided discovery, the therapist and participant work out alternative ways of thinking as well as possibilities to test and transfer these alternative thoughts to real life.
6. *Increasing the use of social and/or professional support in home-based care.* Possibilities of professional and informal help are discussed. The process of allowing and accepting help is discussed using the CBT strategy of cognitive restructuring.
7. *Coping with change, grief, and loss experience.* Central topics are coping with personality changes caused by dementia, the loss of the personal relationship and the resulting feelings of grief. In this context, emotion-based coping-strategies, acceptance of the illness and its resulting changes, as well as experiences of loss are of high importance.
8. *Creating enjoyable positive activities.* Potential incentives are to be increased through the following mechanism: description of the link between positive activities and mood by means of a weekly diary, lists of health-promoting activities, planning of activities, and promoting their implementation.
9. *Improving social skills.* Elements of the social skills training, such as behavioral observation, role-play, and behavioral experiments are performed.
10. *Evaluation.* Changes and goals achieved are summarized and plans for the future discussed.

### Primary outcome measures

*Depressiveness.* A self-developed thermometer scale (0 to 100, vertical) and the Allgemeine Depressionsskala (ADS) [6] will be used to assess depressiveness. The 20 items of the ADS cover emotional, motivational, cognitive, and somatic aspects. Implementation and evaluation are standardized, Cronbach’s *α* is.89.

*Burden of care.* A self-developed thermometer scale (0 to 100, vertical) will be used to assess burden of care.

*Health complaints.* Physical health complaints will be assessed on four domains (fatigue, stomach problems, heart problems and joint pain) by using the Gießener Beschwerdebogen [7]. Besides the values of the four subscales, all 24 items can be used to calculate the subjectively experienced pressure of complaints. Regarding quality criteria, the Gießener Beschwerdebogen has standardized implementation and evaluation procedures, good internal consistency (Cronbach’s *α*.82 to.88 for the subscales and Cronbach’s *α*=.94 for the total scale) and is normed.

*Ability to solve problems relating to focused problem areas.* Problem-solving ability will be measured by Goal Attainment Scaling [8], a non-standardized manual-based instrument providing process-orientated information on how far participants are from reaching individual therapy goals. Caregivers and therapists will rate the goal attainment in the last therapy session.

### Secondary outcome measures

*Anxiety.* Anxiety symptoms will be measured by using the anxiety subscale of the Hospital Anxiety and Depression Scale [9]. This instrument is standardized and normed and has good internal consistency (Cronbach’s *α* is.80 for the anxiety subscale).

*Quality of life.* The WHOQOL-Bref [10] is a standardized and normed questionnaire with 26 items measuring subjective physical and mental well-being as well as satisfaction with social relations and the environment. Cronbach’s *α* is between.77 and.87, implementation and evaluation are standardized. The items of the WHOQOL-BREF will be changed in tense from present to past because the focus is on respondents’ recall of the last two weeks.

*Violence and aggressiveness in caregiving.* Verbal and latent violence and aggressiveness in caregiving will be assessed by a standardized questionnaire assessing the frequency of such behaviors [11]. Cronbach’s *α* for this 7-item instrument is.80.

*Utilization of professional assistance.* An instrument, developed and piloted in a sample of caregiving relatives, will be used to assess utilization of professional assistance in three areas (demand, utilization, and barriers to utilization).

*Cost effectiveness.* The cost-effectiveness analysis will be conducted from the perspective of statutory health insurance with a time horizon of 6 months. This will consist of the costs of the intervention and of the health care utilization of the caregiving relatives. The latter will be assessed by the FIMA questionnaire [12]. In addition, time spent on informal care will be measured by a modified version of the Resource Utilization in Dementia (RUD) questionnaire [13,14]. As a measure of effectiveness, subjectively rated health status of caregiving relatives and quality of life, measured through the EQ-5D [15], will be used.

*Background.* Based on the experience of our previous studies [4,16], a questionnaire will be used, assessing not only the socio-demographic characteristics of carers and people with dementia, but including further aspects. For family caregivers, detailed conditions of the care situation, diseases, sleep quality, physical activity, and medication will be assessed. For the person with dementia, type of dementia, other diseases, and care services used will be assessed.

*Adequateness of 8h training.* The therapists’ rating of adequateness of the 8-hour training program and the supervision will be assessed through a semi-structured interview with each therapist after the end of all therapy sessions.

### Sample size

Based on results of meta-analyses [1,17,18], we can expect interventions for caregiving relatives of people with dementia to have small to medium effects on the mental and physical health of caregivers (*d*=0.14 to 0.41). Results from the previous project [4] similarly indicate that this telephone-based intervention has small to medium effects (*f*=0.18 to 0.29).

Hypothesizing a small effect size in a two-group design with three measurement points, an *α* of.05, a power (1−*β*) of.80, and a *ρ* of.50, we need a sample of *n*=82 per group [19]. Therefore, we aim to have 164 participants in the randomized groups. Assuming a dropout rate of 30%, we plan to recruit 118 persons in each randomized group. Additionally, we aim to recruit as many people for the F2F group as possible (with *n*=118 as cut-off criterion).

### Data analysis

The data will be analyzed with SPSS version 22.0 (IBM Corp., Armonk, NY) and MPlus version 7.0 (Muthén & Muthén, Los Angeles, CA). Data cleaning and analyses will be performed using SPSS/Mplus syntax operations. Possible baseline differences between trial arms will be statistically tested and further analyses will be adjusted for imbalances in the baseline scores. The primary analysis will be by intention to treat. For the primary outcomes, analyses will be corrected for multiple testing.

As sensitivity analyses, completers of all assessments will be analyzed separately. Hypotheses tests will be performed at *α*=.05. The numeric outcomes will be analyzed using analyses of variance; categorical outcomes will be analyzed using *χ*^2^-tests. Additionally, latent change model analyses will be performed for continuous longitudinal outcome data. To handle missing data, full information maximum likelihood estimation will be conducted in MPlus. As part of the questionnaire adaptation, factor, reliability, and correlation analyses will be performed.

## Discussion

This study will evaluate whether TEL improves depressiveness, burden of care, health complaints, and problem-solving ability compared to usual care and assess whether TEL is as effective as F2F. One strength of this study is its robust design and follow-up assessment allowing evaluation of the long-term effects of TEL. For the first time, it will be possible to test intervention effects of TEL in established care services.

However, there will also be several challenges in this study. In particular, enrolling a sufficiently large sample in the F2F group will be a major difficulty, which we hope to overcome by recruiting via different media and by directly assigning participants to F2F.

The F2F sample will be especially selective, as it will consist of an exclusively urban and mobile population. Since possible F2F participants will be selected from the overall recruited sample, the remaining subsample being randomized will potentially be more rural and less mobile than the original sample recruited.

To ensure adequate and homogeneous application of therapeutic strategies, extensive training prior to implementation, application through CBT-qualified therapists, regular supervision (4 to 6 times per year) and documentation of the implementation will be essential strategies. To determine treatment integrity, independent CBT-qualified therapists will assess psychotherapeutic competence and adherence in randomly selected audio and video recordings of the sessions.

In case the intervention is terminated prematurely, an attempt will be made to continuously include the participant in the data collection. To avoid selective dropout in the control group, this group will receive an incentive. Blinding is not possible in the design of the proposed study, since both the family caregivers and the therapists will know whether a therapy is conducted. To nevertheless guarantee an independent assessment of intervention efficacy, the data collection will be performed by researchers who are not involved in the therapy.

In summary, this will be the first study to test the implementation of telephone-based cognitive-behavioral therapy in established care provision structures in a randomized controlled trial. If TEL proves to be effective in improving primary outcomes, our results will be relevant to practice. Hence, TEL could be used on a large scale. Thus, the results of the study will be used to improve support services for family-caregivers.

## Abbreviations

TEL: Telephone-based cognitive-behavioral therapy
F2F: Face-to-face cognitive-behavioral therapy
CBT: Cognitive-behavioral therapy
Tele.TAnDem: Cognitive-behavioral telephone-based intervention for family caregivers of people with dementia
ADS: Allgemeine Depressionsskala
